# Differential Nucleosome Occupancies across Oct4-Sox2 Binding Sites in Murine Embryonic Stem Cells

**DOI:** 10.1371/journal.pone.0127214

**Published:** 2015-05-18

**Authors:** Amy Sebeson, Liqun Xi, Quanwei Zhang, Audrey Sigmund, Ji-Ping Wang, Jonathan Widom, Xiaozhong Wang

**Affiliations:** 1 Department of Molecular Biosciences, Northwestern University, Evanston, Illinois, United States of America; 2 Department of Statistics, Northwestern University, Evanston, Illinois, United States of America; Michigan State University, UNITED STATES

## Abstract

The binding sequence for any transcription factor can be found millions of times within a genome, yet only a small fraction of these sequences encode functional transcription factor binding sites. One of the reasons for this dichotomy is that many other factors, such as nucleosomes, compete for binding. To study how the competition between nucleosomes and transcription factors helps determine a functional transcription factor site from a predicted transcription factor site, we compared experimentally-generated in vitro nucleosome occupancy with in vivo nucleosome occupancy and transcription factor binding in murine embryonic stem cells. Using a solution hybridization enrichment technique, we generated a high-resolution nucleosome map from targeted regions of the genome containing predicted sites and functional sites of Oct4/Sox2 regulation. We found that at Pax6 and Nes, which are bivalently poised in stem cells, functional Oct4 and Sox2 sites show high amounts of in vivo nucleosome displacement compared to in vitro. Oct4 and Sox2, which are active, show no significant displacement of in vivo nucleosomes at functional sites, similar to nonfunctional Oct4/Sox2 binding. This study highlights a complex interplay between Oct4 and Sox2 transcription factors and nucleosomes among different target genes, which may result in distinct patterns of stem cell gene regulation.

## Introduction

The binding of transcription factors to DNA is a critical step in the regulation of gene expression. Transcription factors have specific DNA binding motifs [1, 2], which are found millions of times within the genome. However, functional binding sites, identified by occupancy in ChIP experiments, occur with much less frequency. While much work has been done to identify binding motifs for transcription factors as well as to identify binding affinities in vitro [3–6], these two inputs are not enough to predict whether a binding site is actually utilized in vivo [3, 7]. Many other factors are involved in this process, such as cooperativity between transcription factors [8, 9], both direct and indirect, as well as competition between other transcription factors [10, 11] or other DNA-binding proteins such as nucleosomes and other chromatin binding proteins [12, 13].

As 75–90% of the eukaryotic genome is bound by histone proteins [14], understanding how nucleosomes and transcription factors compete for DNA is one important aspect of the process for forming a functional transcription factor binding site (TFBS). In vitro studies have examined this competition at its simplest. When a TFBS is located in the exact center of a nucleosome favoring sequence, the probability of that TF outcompeting histone proteins is very small. However, as the TF site is moved towards the ends of the nucleosome favoring sequence, the TF is then able to compete with the histone proteins and bind with increasing probability [15, 16]. Thus, the location of nucleosome-forming (or nucleosome-disfavoring) sequences relative to TF binding sequences is likely to be a critical factor in determining functional TFBS in vivo.

While this competition plays out in a straightforward manner in vitro, the influence of this competition is harder to evaluate in vivo. Techniques such as ChIP-seq and micrococcal nuclease (MNase) mapping of nucleosomes [17–26] have provided a wealth of information regarding the position of transcription factors and histone proteins in vivo. However, it is difficult to extract the influence of the competition between TFs and nucleosomes since these techniques measure the final outcome of the binding process. To assess the role of competition with nucleosomes in establishing functional TFBS, the spacing between TF binding sequences and nucleosome disfavoring sequences was manipulated in a yeast promoter. Different distances between these two elements resulted in changes in nucleosome positioning, which altered TF binding and gene expression levels [27], illustrating that nucleosomes can clearly compete against TFs. While it would be very difficult to carry out this type of experiment in a higher eukaryote, other approaches can be used to study nucleosome-TF competition and its role in regulating gene expression.

One of the issues when examining nucleosome competition genome-wide in higher eukaryotes is the need to understand where nucleosome favoring or disfavoring sequences are located and their potential strength. Thus, to better understand this process in vivo, one needs to determine the intrinsic preference of a nucleosome for any particular stretch of DNA sequence, which we refer to as nucleosome preferences. These preferences can be measured by generating in vitro nucleosome maps, where chromatin is reconstituted from genomic DNA and histones and then digested with MNase [28]. Since the only factors present are histones, the DNA bound in this experiment represents the nucleosome preferences that are inherent to the underlying DNA sequence. Thus, by comparing the nucleosomes’ preferred binding locations with potential transcription factor binding sites, we should have a more nuanced understanding of how nucleosome competition helps to determine a functional TFBS.

In yeast, in vitro nucleosome experiments have been performed and have demonstrated that nucleosomes have preferred binding patterns of DNA sequence [28, 29]. Furthermore, work in Kaplan *et al* analyzed the results of in vitro nucleosome maps in yeast and created an algorithm to predict nucleosome binding preferences for any stretch of DNA [29]. Many have used this predictive algorithm to examine correlations between nucleosome occupancy and transcription factor binding [29–34]. One of the concerns with the conclusions from these experiments is that they rely on the predictive algorithm rather than examining in vitro nucleosome occupancy experimentally. This algorithm was derived from yeast DNA, and while it is highly predictive of in vivo nucleosome occupancy in yeast, it was only moderately predictive for in vivo nucleosome occupancy in species other than yeast [29, 31]. This suggests that the algorithm may not be appropriate for capturing nucleosome preferences in higher eukaryotes and that experimentally derived in vitro nucleosome occupancies should provide more precise data. Additionally, while past experiments have addressed the patterns of nucleosome occupancy surrounding functional TFBS (i.e. ChIP-seq binding sites), none have examined the differences between predicted transcription factor binding sites and functional sites to determine if the observed patterns are unique to functional TFBS.

Therefore, we aimed to study the relationship between nucleosome preferences and both predicted and functional TFBS using the first experimentally-derived in vitro nucleosome map from murine embryonic stem cells, as well as a control in vivo nucleosome map. Part of the difficulty in obtaining nucleosome occupancies from more complex organisms, either in vivo or in vitro, is due to the large size of the genome and consequently lower sequencing coverage per bp. To overcome this challenge, a BAC solution-hybridization technique was modified and developed in our lab to enrich genomic pools of mononucleosomes [35]. We performed genomic reconstitutions of mouse ES cell DNA and digested the reconstituted chromatin to obtain in vitro nucleosome fragments. We also digested ES cells with MNase to obtain in vivo nucleosomes. These samples were processed with the BAC-based enrichment technique, selecting 6 target promoter regions to examine in detail: Nestin, Oct4, Olig2, Pax6, Sox1 and Sox2. Within these promoters, we identified possible binding sites of the transcription factor network of Oct4 and Sox2, which controls the cell fate decision between pluripotency and differentiation in stem cells [36–40] and confirmed the functionality of these binding sites using published ChIP-seq data [37]. [41, 42].

Our experimental approach allowed us to examine both in vivo and in vitro nucleosome occupancy with much higher sequencing coverage than genome-wide mapping [43]. By combining a detailed, experimentally-generated profile of nucleosome sequence preferences with a comparison of both predicted and functional TFBSs, our results suggest that Oct4 and Sox 2 transcription factors can decrease nucleosome occupancy in vivo at poised genes, but do not alter in vivo nucleosome occupancy at active genes.

## Materials and Methods

### Embryonic stem cell culture

R1 embryonic stem cells, obtained from the Nagy Lab at the ES cell core facility at the Samuel Lunenfeld Research Institute Mount Sinai Hospital, were cultured as previously described [44]. Cells were cultured on 0.1% gelatin treated plates in feeder-free conditions with embryonic stem cell media containing LIF (DMEM (Invitrogen 11965–118), 15% FBS (Millipore ES-009-B) 1x Non-Essential Amino Acids (Invitrogen 11140–050), 1x Sodium Pyruvate (Invitrogen 11360–070), 1x L-Glutamine (Invitrogen 25030–081), 100μM Beta mercaptoethanol (Sigma M7522-100ML), 1x Penicillin/Streptomycin (Invitrogen 15140–122), 10^3^ U/mL LIF (Millipore ESG1106)).

### In vivo mononucleosome purification

Cells were permeablized while adherent to culture dishes with lysis buffer (10mM Tris-Cl (pH 7.4), 10mM NaCl, 3mM MgCl_2_, 0.5% NP-40, 0.15mM spermine, 0.5mM spermidine). Chromatin was then digested with micrococcal nuclease (Sigma N3755) at a concentration of 1000 Worthington units of MNase per 100 mm culture plate in digestion buffer (10mM Tris-Cl (pH 7.4), 15mM NaCl, 60mM KCl, 0.15mM spermine, 0.5mM spermidine, 1mM CaCl_2_) for 30–60 minutes at room temperature. Digestion was stopped with 1/10 volume stop solution (0.25M EDTA, 5% SDS). DNA was isolated via phenol/chloroform extraction and ethanol precipitation.

Mononucleosomal DNA was isolated by running digested DNA on a 3.3% NuSieve (Lonza 50084) gel at 75 V for 5 hours. DNA of 147 bp in length was excised from the gel and purified using a crush and soak technique. The gel slice was crushed with a small pestle inside a 15mL conical tube, then was covered with crush and soak buffer (300mM NaOAc and 1mM EDTA (pH 8.0)) and incubated with gentle rotation for 48 hours. The solution was spun at 5000xg for 1 hour, after which the solution was filtered using low-bind centrifuge filters (Millipore UFC40VV00). This solution was then purified using a QIAquick PCR purification reaction (Qiagen 28104). Finally, ABI SOLiD library preparation was performed according to manufacturer’s recommendations (Invitrogen 4464411), to obtain adaptor-ligated mononucleosomal DNA.

### Genomic DNA purification and in vitro reconstitution of chromatin

Embryonic stem cells were cultured as described above, collected via trypsinization with 0.05% trypsin (Invitrogen 25300–054), inactivated with serum-containing media, then combined for a total of ~5x10^7^ cells. Cells were resuspended in extraction buffer (10mM Tris, 0.1M EDTA, 0.5% SDS). RNA was removed by incubating in 20μg/mL RNaseA at 37°C for 1 hour. Protein was subsequently removed by incubating with 100μg/mL Proteinase K for 3 hours at 50°C. The solution was phenol extracted four times, placed into a 3.5 kDa, 50mm dialysis tube, and dialyzed three times in 4L of dialysis buffer (50mM Tris, 10mM EDTA). After dialysis, the solution was isopropanol precipitated, and the resulting genomic DNA was resuspended in 1x TE.

To obtain histone octamer for in vitro reconstitutions, chicken erythrocytes were prepared as described previously [45]. Reconstitutions were assembled using a mass ratio of 30μg histone octamer to 100μg genomic DNA in reconstitution buffer (2M NaCl, 5mM Tris, 1mM benzolamide, 0.5mM PMSF, 0.5mM EDTA) and placed inside 12-14kDa, 10mm diameter dialysis tubing. Each reconstitution tube was then placed in a 6-8kDA 100mm dialysis bag filled with 100mL of reconstitution buffer. This bag was then dialyzed 3 times in 4L of dialysis buffer (5mM Tris, 1mM benzolamide, 0.5mM PMSF, 0.5mM EDTA).

### In vitro mononucleosomal DNA purification

Reconstitution mixture was suspended in MNase buffer (10mM Tris-Cl (pH 7.4), 15mM NaCl, 60mM KCl, 0.15mM spermine, 0.5mM spermidine, 1mM CaCl_2_) and digested with 5U of MNase per 10μg of genomic DNA present for 5 min at 37°C. Digestion was stopped using 1/5 vol. 0.125M EDTA, and samples were purified via phenol/chloroform extraction, and ethanol precipitation. Samples were run on a 5% native acrylamide gel at 175V for 2 hours. DNA of 147bp was excised from the gel and purified using a crush and soak technique, as described above. ABI SOLiD library preparation was then performed (Invitrogen 4464411) to obtain adaptor-ligated DNA.

### Bacteria artificial chromosome preparation

BAC clones of specific mouse genomic regions were selected by hand and ordered from the RPCI-23/24 library from Children’s Hospital Oakland Research Institute, Oakland, CA, USA. Clones were streaked on LB-CA selection plates, individual colonies were picked and grown in 5mL LB-CA liquid culture for 8 hours at 37°C while shaking, then larger preparation cultures were grown by adding 0.5mL of the starter culture to 100mL of liquid LB-CA and incubating while shaking at 37°C for 16 hours. Cells were then spun for 20 minutes at 5000xg and resuspended in 10mL P1 buffer (50mM Tris-Cl, pH 8.0, 10mM EDTA, 100μg/mL RNase A). Gentle lysis was performed by adding 10 mL P2 buffer (200mM NaOH, 1% SDS) and incubating for 5 min. Finally, the solution was neutralized via addition and vigorous mixing of 10mL P3 buffer (3.0M potassium acetate, pH 5.5). This mixture was then purified according to the Qiagen 100-G Genomic Tip protocol (Qiagen 12143) and isopropanol precipitation.

After clone purification, each BAC was accurately quantified, combined into batches with equal molecular weight so that the total mass of the batch was 200ng. Each batch was then nicked to add biotin tags, using Roche’s nick translation kit (Roche 10976776001) and 40μM biotin-dUTP (Axxora ENZ-42811). The reaction was assembled per kit instructions, incubated for 1 hour at 16°C, then purified using a spin chromatography column (Bio-Rad 732–6223).

### Solution hybridization enrichment

100ng of biotin-nicked BAC DNA was concentrated and resuspended with 10μg of Cot-1 DNA (Invitrogen 18440–016). This mixture was denatured, suspended in 2x hybridization buffer (1.5 M NaCl, 40 mM sodium phosphate buffer (pH 7.2), 10 mM EDTA (pH 8), 10x Denhardt’s, 0.2% SDS), then incubated at 65°C for 6 hours. 1μg of adaptor-ligated mononucleosome DNA was denatured, also suspended in 2x hybridization buffer, then combined with the BAC DNA mixture and incubated at 65°C for a further 70–80 hours. To isolate the enriched mononucleosomal DNA, the hybridization solution was bound to 100μL of washed streptavadin-coated beads (Invitrogen 11205D) suspended in 150μL of washing/binding buffer (10 mM Tris-HCl (pH 7.5), 1 mM EDTA (pH 8) and 1 M NaCl) incubated for 30 minutes, then stringently washed with one wash of 1x SSC, 0.1% SDS buffer at room temperature, and three washes of 0.1x SSC, 0.1% SDS buffer at 65°C. The DNA was eluted with 100μL of 0.1 M NaOH, neutralized with 100μL of 1M Tris-HCl (pH 7.5), then desalted and cleaned using two spin chromatography columns (Bio-Rad 732–6223).

Single-stranded enriched DNA was then amplified and isolated using a high-fidelity polymerase kit (NEB E0553S) according to recommended reaction conditions, and using ABI SOLiD library primers (Invitrogen 4464411). Test reactions of 25μL were cycle titrated to determine the appropriate number of rounds of amplification, then bulk reactions of 2x 50μL were amplified. These products were loaded onto a 3.3% NuSieve agarose gel in TAE and excised and purified using the previous outlined crush and soak method. The DNA was then quantified and sent for next-generation DNA sequencing.

### DNA sequencing and mapping

DNA fragments were mapped using ABI SOLiD paired-end technology. The data was converted from colorspace and aligned to the genome using bowtie alignment packages [46]. Alignment was completed using step-wise mismatch settings, allowing 0 mismatches, then allowing 1 mismatch on all non-aligned reads, etc. up to 3 mismatches. Nucleosome centers were directly calculated by dividing the length of the sequencing read in half, and nucleosome occupancy was determined by applying a Gaussian weight to the center of the nucleosome position. If a sequence length is odd, a Gaussian weight of exp [-0.5*(*d*/20)^2^] is assigned to a position *d* bp away from the center of the sequence for *d* ≤ 73. If a sequence length is even, then the central two positions were treated as the center in turn to assign a weight exp [-0.5*(*d*/20)^2^] for a position *d* bp away from the center for *d* ≤ 73. To normalize the center-weighted occupancy scores, the average occupancy of each dataset was calculated, then individual bp scores were divided by the average to normalize the occupancy.

### GC sequence bias correction

Finally, occupancy was adjusted for GC content to account for potential sequence bias. Others have shown that GC content can be highly correlated to nucleosome occupancy in yeast and that MNase may lead to enrichment of GC-rich sequences [47]. A micrococcal nuclease map of naked DNA can be used to correct for this bias. Genomic yeast DNA was digested with MNase and sequenced with paired-ends [48]. Reads were aligned to sacCer2 with bowtie [46], using step-wise mismatch alignment as described above. Using only uniquely aligned genes, the read coverage for each bp was divided by the average read coverage per bp for the genome to give normalized read coverage data.

The *S*. *cerevisiae* genome was divided into 20 bp bins and the normalized average reads score (*S)* and the G/C percentage of each bin (*GC)* was calculated, resulting in ~603K pairs (*S*, *GC)*. Based on a scatter plot of all (*S*,*GC*), a quadratic regression of best fit was calculated for *log(S)*,*GC*, denoted as *F(GC)*.

To adjust the BAC enriched data, the G/C percentage for each position *i* was calculated using a +/- 10 bp window (*GC*_*i*_), then *F(GC*_*i*_ *)* was calculated. The normalized, center-weighted occupancy score for each bp (*S*_*i*_) was then divided by exp(*F(GC*_*i*_ *)*) to give non negative, GC adjusted center-weighted occupancy scores. Values < = to 0.01 were set to 0.01.

### Transcription factor site identification and occupancy calculations

Predicted transcription factor binding sites were identified using the matrix for Oct4/Sox2 co-binding from the JASPAR database [49] and scanning the regions surrounding the six gene regions as described (Figure C in S1 File). The 58 identified sites were then classified as either functional or nonfunctional, based on an average TF occupancy score greater than 20 from Whyte *et al*. [37]. In vivo and in vitro occupancy for each TFBS was calculated by averaging the adjusted, center-weighted score over the 15 bp of the site. The average occupancy and average fold-change were then calculated for each class of gene and for functional and nonfunctional sites, using the standard error of the mean to plot error. Significance between functional and nonfunctional averages and class averages was determined using a paired z-test. The fold-change at each site was calculated by taking the log base 2 of the ratio of the average in vivo occupancy to the average in vitro occupancy. The median was examined for the distribution of fold-change results, since small in vitro scores result in large fold values that strongly influence the average.

## Results

### Enriching nucleosome populations with solution hybridization to bacterial artificial chromosomes

**Fig 1:**
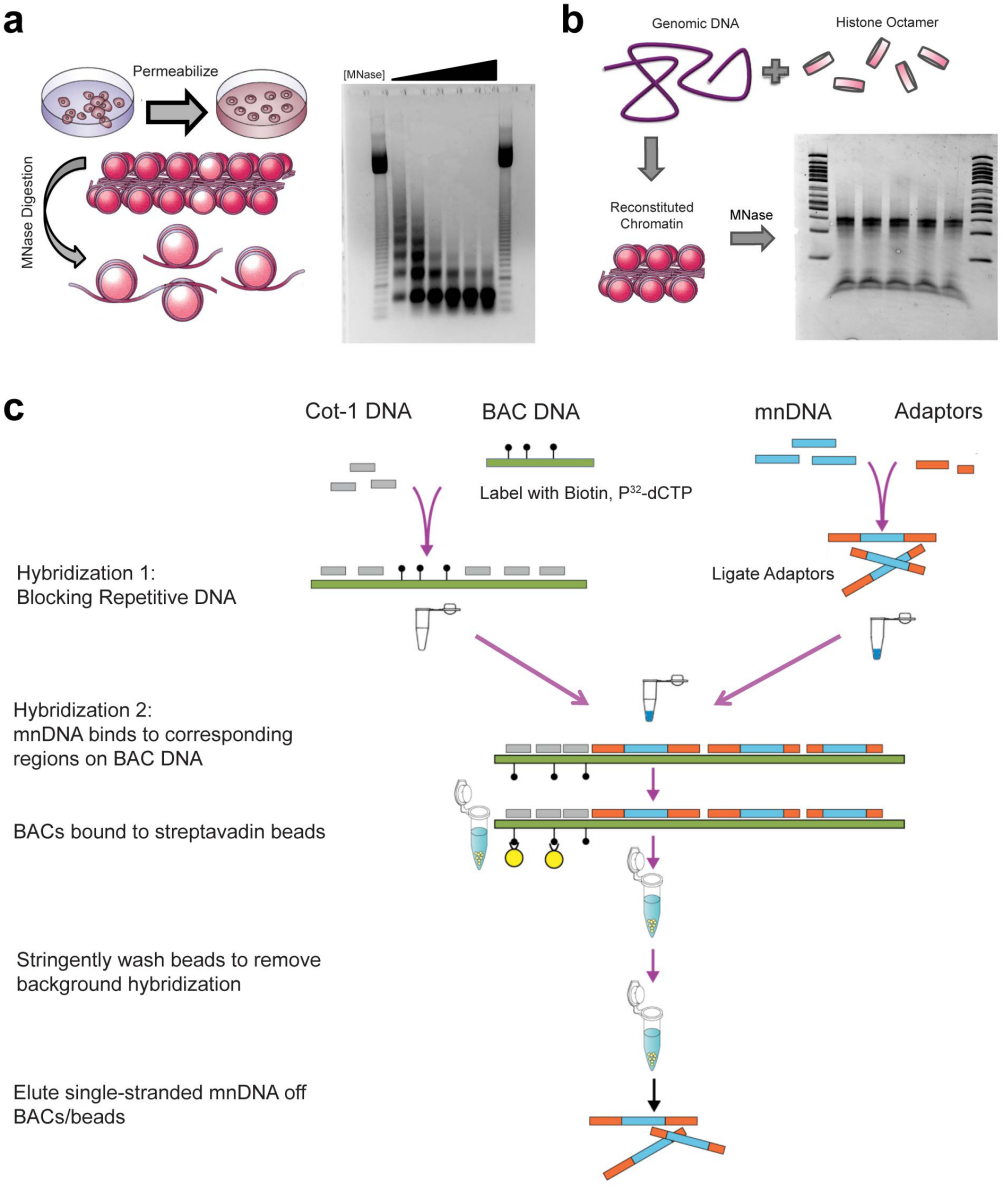
Mapping in vivo and in vitro nucleosome occupancy using BAC-based enrichment. (a) Protocols were modified so that permeabilization and micrococcal nuclease digestion occur while embryonic stem cells are attached to the tissue culture surface to improve recovery and digestion reproducibility. The amount of micrococcal nuclease (MNase) in the digestion was titrated so that the mononucleosome band at 147bp is the primary band, without overdigestion. Digests were measured in Worthington Units of MNase * Time of digestion / volume of cell culture (U*min/mL). Lanes 1 and 8: 50bp ladder. Lanes 2–7 range from 2500 U*min/mL to 25,000 U*min/mL of MNase, with ideal digestion in the third condition, 10,000 U*min/mL. This amount of digestion was used in all future experiments. (b) Genomic DNA was purified from embryonic stem cell cultures and combined with histone octamer purified from chicken erythrocytes in a ratio of 100μg:30μg under high salt (2M NaCl) conditions. Removal of salt via dialysis results in reconstituted chromatin, representing histone proteins’ preferred DNA sequences. Reconstituted chromatin was digested with 5 Worthington Units of micrococcal nuclease per 10μg of genomic DNA present, for a digestion of 5 minutes at 37°C (Lanes 1 and 7: 50 bp ladder; Lanes 2–6: digested chromatin). (c) Bacterial Artificial Chromosome (BAC) DNA, which was nicked with biotin-dUTP, was blocked with Cot-1 DNA at a ratio of 100ng:10μg. 1μg of library-adapted mononucleosome DNA was denatured and mixed with BAC DNA. Mononucleosome DNA was hybridized to the corresponding BAC region and was isolated by removing BACs from solution with streptavadin beads, stringently washing the beads, and eluting single stranded DNA from the beads. Double stranded products were amplified using PCR and sent for paired-end sequencing.

To obtain in vivo nucleosome positions for ES cells, mouse ES cells [44] were cultured under feeder-free conditions. Immunofluorescent staining confirmed that clusters of ES cells were uniformly positive for the stem cell marker SSEA1 (Figure A in S1 File). Meanwhile, measurement of the nucleosome repeat length verified a value of 187 bp for these cells, which agrees with the range of values in the literature [17, 21, 43], suggesting that ES cells maintained the pluripotent chromatin state on a global scale (Figure B in S1 File). Mononucleosomal DNA was prepared for in vivo nucleosome mapping by directly digesting these cells adherent in culture dishes with MNase (Fig 1a).

To assay in vitro nucleosome positioning signals, we performed in vitro reconstitutions of chromatin using genomic DNA isolated from ES cells. By adding a low ratio of histone octamer to genomic DNA (3:10), nucleosomes are not positionally constrained and are able to sample DNA for the most energetically favorable sequences. These reconstitutions were digested with MNase to obtain a pool of DNA that represents in vitro sequence preferences (Fig 1b).

The solution-based hybridization technique of Yigit *et al*. [35], known as BEM-seq, was adapted to generate enriched nucleosome maps from the pool of linker-ligated mononucleosomal DNA. We selected 6 bacterial artificial chromosomes (BACs) from Children’s Hospital Oakland Research Institutes RPCI23/24 library (Table 1). These BACs contained the full promoters and gene regions of Oct4 (Pou5f1) and Sox2, which are master regulators of pluripotency that also self-regulate their own expression [36], as well as Sox1, Nes, Pax6, and Olig2, which are developmental regulators that are regulated by bivalent poised promoters [41, 42]. In BEM-seq experiments (Fig 1c), purified BACs were labeled with dUTP-biotin by nick translation, and hybridized with the nucleosome pools in the presence of Cot-1 DNA to remove repetitive sequences. After hybridization, the BAC-hybridized, nucleosomal DNA was enriched by streptavadin-coated magnetic beads and amplified by PCR.

**Table 1:** Selected BAC enrichment regions.

BAC Name	Gene Name	Chromosome	BAC Start	BAC End	BAC Length (bps)
RP24-379E2	Nes	chr3	87694297	87864718	170422
RP23-285O8	Olig2	chr16	91182647	91321416	138770
RP24-247A9	Pax6	chr2	105457424	105604145	146722
RP24-245P6	PouF51	chr17	35562956	35713379	150424
RP24-238C10	Sox1	chr8	12352075	12508633	156559
RP24-298D8	Sox2	chr3	34443631	34605476	161846

6 BAC regions were selected from CHORI’s RP23/24 library. They were selected to contain the promoter regions of bivalent genes not regulated by Oct4/Sox2 (Olig2,Sox1), bivalent genes regulated by Oct4/Sox2 (Nes and Pax6), and genes activated by Oct4/Sox2 (PouF51 and Sox2).

To estimate the efficiency of the enrichment, a portion of the selected DNA was cloned, and individual clones were analyzed via conventional sequencing. Using the Oct4 locus as an example, we calculated the probability of sequencing a nucleosome from the region in an unenriched pool by dividing the number of bps of the BAC region by the number of bp in the genome. For this locus, the value corresponds to p = 5x10^-5^. In the enriched pool, we directly calculate the probability of sequencing an enriched nucleosome from the region by dividing the number of clones targeted to the region by the total number of clones sequenced, which corresponds to p = 0.64. The ratio of enriched to unenriched probabilities corresponds to the fold level of enrichment, which for this locus was 12,800-fold.

### BEM-seq generates high-coverage, local nucleosome maps

**Fig 2:**
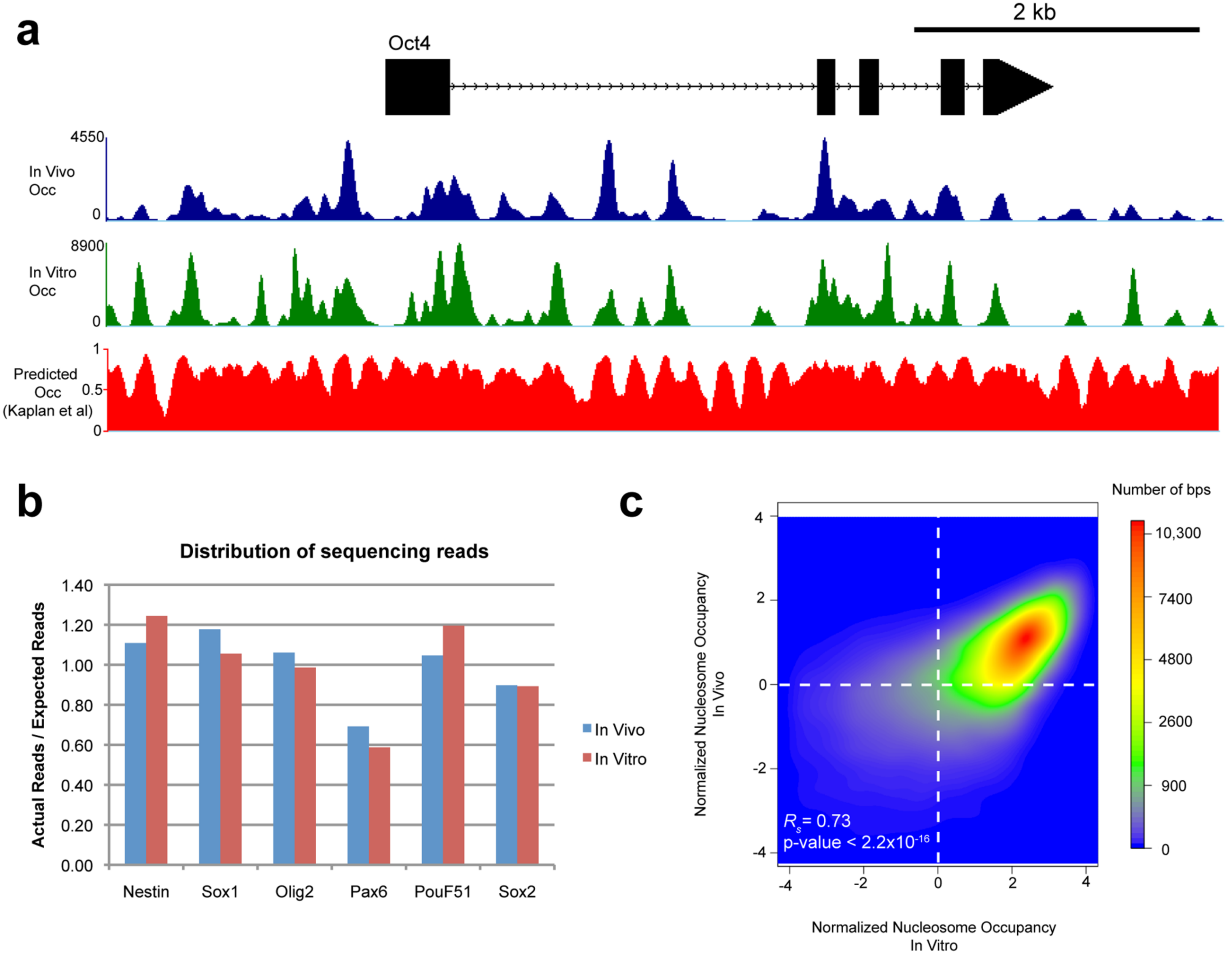
BEM-seq generated nucleosome occupancies are reproducible and reveal correlation between In vivo and in vitro occupancy. (a) Tracks of nucleosome occupancy are displayed across the Pou51f (Oct4) gene. In vivo nucleosome occupancy is represented in blue, and in vitro nucleosome occupancy is represented in green. Occupancy is center-weighted but not normalized, representing the amount of sequencing reads present in the experiment. Predicted in vitro occupancy, calculated as described in Kaplan *et al*, is also shown. Predicted in vitro data is uniformly weighted and displays the probability of in vitro nucleosome occupancy for a given bp from 0 to 1. (b) The sequence read coverage of each BAC is shown. The percentage of on target reads is calculated by taking the actual number of reads per BAC and dividing by the expected number of reads per BAC (The number of total sequencing reads multiplied by the ratio of the BAC length in bp to the total experiment size). Each BAC shows similar levels of enrichment from in vivo and in vitro experiments. (c) A density scatter dot plot of in vivo versus in vitro normalized occupancy scores is shown. For each sample, the log_2_ of the normalized occupancy is taken, and the genome average is subtracted to set the average plotted value to zero. In vivo and in vitro nucleosome occupancy tightly correlate with each other. Spearman-rank correlation analysis for all base pairs in the datasets confirms the similarity between the two datasets (0.73, p <2.2x10^16^). This reflects the influence of intrinsic nucleosome preferences on in vivo occupancy.

To derive nucleosome maps, the in vivo and in vitro enriched nucleosome fragments were sequenced using ABI’s SOLiD paired-end sequencing platform (Table 2). We aligned the sequencing reads to the mouse reference genome (mm9) using Bowtie (http://bowtie-bio.sourceforge.net/index.shtml) by allowing up to two mismatches. Only the uniquely mapped reads were kept to calculate the center-weighted nucleosome occupancy score at each bp in the BAC regions (Fig 2a, see Materials and Methods). Compared to an average coverage of 20 reads per bp in an existing genome-wide nucleosome map of murine ES cells [43], our in vivo and in vitro maps present significantly better coverage with up to 4500 and 9000 reads per bp respectively in targeted BAC regions. The improved sequencing coverage allows us to better resolve nucleosome positions for downstream analysis. Compared to a computationally-derived predicted nucleosome occupancy map [29], we found that our experimentally-derived in vitro map provides better resolution of nucleosome positions, shown by sharper nucleosome centers and more dynamic range between nucleosome-covered and nucleosome free regions. 87% of uniquely mapped reads were from the targeted BAC regions, demonstrating the specificity of our hybridization enrichment protocol.

**Table 2:** ABI SOLiD sequencing results of in vivo and in vitro nucleosome occupancy maps.

	In Vivo	In Vitro
Total ends	49.75 M	70.17 M
Paired reads	20.39 M	29.85 M
% paired	40.99%	42.54%
Unique reads	16.59 M	25.29 M
% unique	81.33%	84.71%
# of BACs	6	6
Total bps	924,743	924,743
Reads/bp	3587	5470

We analyzed the results of the sequencing run in the following manner. First, we defined paired reads for pairs where each end aligned to the same chromosome. From that subset, we removed alignment scores that were not unique as assigned by bowtie. Finally we estimated the sequencing coverage (Reads/bp) by multiplying the number of reads by the average length in bp of each read, then dividing by the number of bps each BAC pool contained.

To compare the in vivo and in vitro maps, we first calculated the ratio of actual over expected number of reads mapped to each BAC (the expected number was obtained by distributing the total number of sequencing reads proportionally, according to each BAC length). Fig 2b shows that the in vitro and in vivo ratios are consistent to a large extent for any given BAC, suggesting that the BAC enrichment technique is highly replicable. The variation of the ratio statistics across different BACs may reflect differences in DNA sequence features that affect the intrinsic nucleosome occupancy or sequence bias, which may affect the hybridization reaction, MNase digestion, or sequencing. Finally, we compared the in vivo and in vitro maps using Spearman’s correlation analysis. The occupancy scores from the in vivo and in vitro map have a Spearman correlation of 0.73 (Fig 2c, shown are occupancy scores normalized by average occupancy in log scale), which suggests that in vitro preferences influence the in vivo nucleosome occupancy.

### Examples of nucleosome organization at functional and predicted Oct4/Sox2 sites

**Fig 3:**
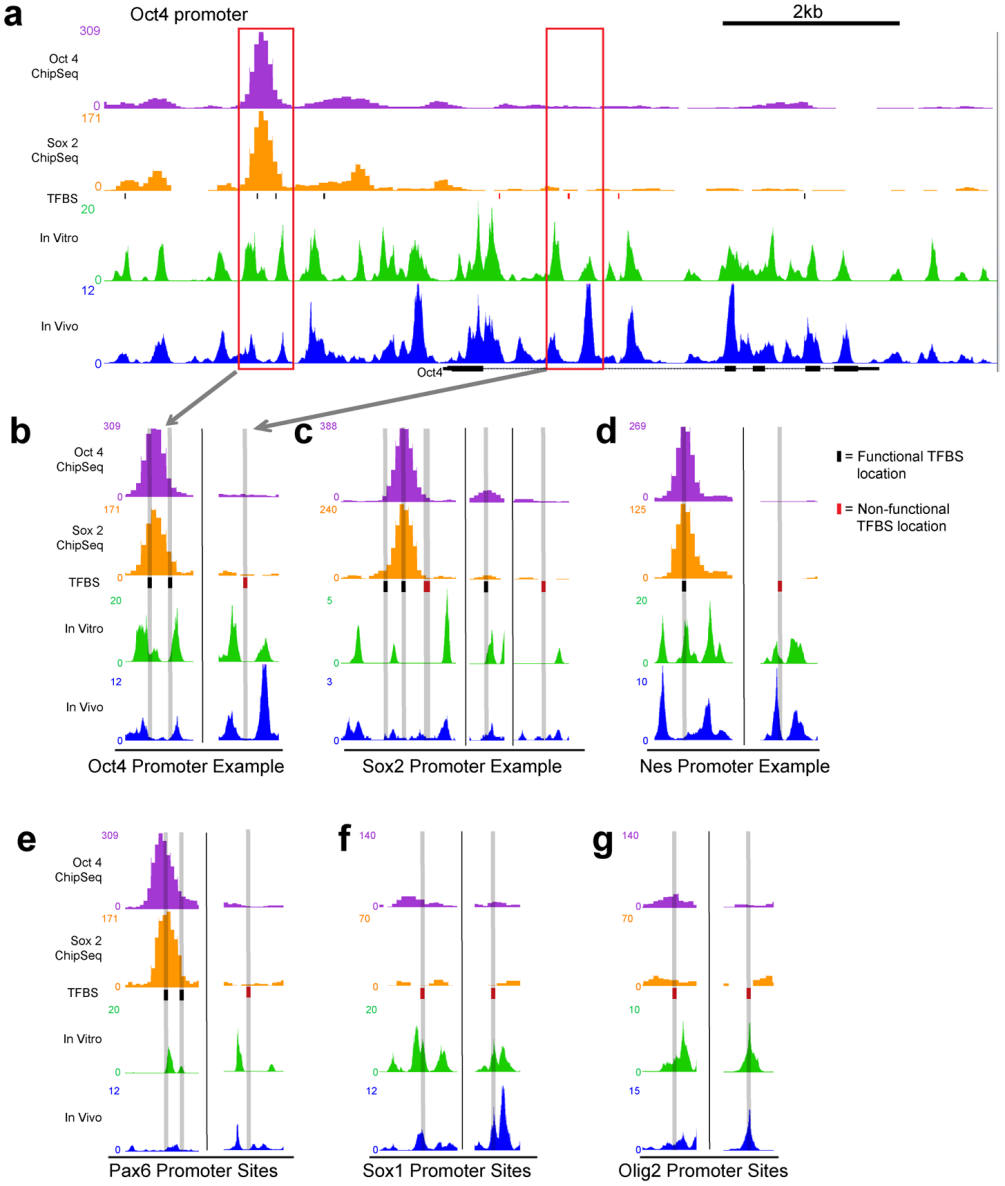
In vivo and in vitro nucleosome occupancy tracks at functional and non functional transcription factor binding sites. (a) A 10kb region containing the Oct4 (Pou5f1) gene locus is shown with tracks for Oct4 occupancy and Sox2 occupancy [37], as well as normalized, GC-adjusted in vitro nucleosome occupancy and in vivo nucleosome occupancy. The TFBS track contains predicted binding sites, generated by the Oct4:Sox2 binding matrix from JASPAR database, with functional sites in black and nonfunctional sites in red. The horizontal scale from Fig 3a is maintained for all subsequent figures. For each gene in our experiment, we chose example regions to examine the patterns of nucleosome occupancy over functional and nonfunctional sites. We highlight the selections for Oct4 to be shown in detail in Fig 3b. (b) Two regions from the Oct4 promoter region are shown. The region in the left panel contains two functional TFBSs. The overlap between the TFBS and the occupancy tracks is displayed with a grey vertical bar across all tracks. In the case of the Oct4 gene, in vivo and in vitro occupancy overlap at both functional and nonfunctional binding sites. (c) Two regions from the Sox2 promoter are shown in detail. Like Oct4, in vivo and in vitro occupancy are colocalized. (d-e) Unlike Oct4 and Sox2, Nes and Pax6 are poised, and show low in vivo occupancy at functional sites, compared to in vitro occupancy. (f-g) Sox1 and Olig2 are not regulated by Sox2 or Oct4, and consequently do not contain any functional TFBS. In vivo and in vitro occupancy seem similar at these nonfunctional sites.

Using the high-resolution local nucleosome maps generated by BEM-seq, the relationship between nucleosome occupancy and TFBS in the mouse genome was examined. We chose to focus our analysis on TFBSs for the Oct4/Sox2 transcriptional network in ES cells. This network uses Oct4 and Sox2 to simultaneously regulate pluripotency targets, which includes their own self-regulation, and developmentally poised genes to be turned on during the process of differentiation [36]. To identify all potential Oct4/Sox2 binding sites within the genes of interest, we scanned the promoter and downstream regions of Oct4, Sox2, Pax6, Nes, Sox1 and Olig2 for predicted factor sites using the 15 bp affinity matrix from JASPAR [49] for Oct4 and Sox2 concurrent binding (Figure C in S1 File). A total of 58 predicted sites were identified (Table A in S1 File). To determine whether predicted TFBS were functional, we examined ChIP-seq data for Oct4 and Sox2 from Whyte *et al*. [37]. The occupancy scores for Oct4 and Sox2 were very highly correlated (R = 0.98, *p* < 2.2e-16), thus we averaged both transcription factors’ occupancy to find overall average TF occupancy at each site and defined functional sites as those with occupancy scores > = 20. 10 out of 58 predicted sites were identified as functional TFBS. An example of these in vivo and in vitro nucleosome occupancies across the Oct4 promoter was visualized in the UCSC genome browser (Fig 3), and the differences of occupancy between nonfunctional Oct4/Sox2 sites and functional ones were examined in more detail (Fig 3b–3g).

We divided the six genes we examined into three functional classes: Class 1 genes (Oct4, Sox2), which are regulated by Oct4/Sox2 and are transcriptionally active [36], Class 2 genes (Pax6, Nes), which are regulated by Oct4/Sox2 and are poised for transcription [42], and Class 3 genes (Sox1, Olig2), which are not regulated by Oct4/Sox2 but are bivalent [42]. We then visually compared in vitro and in vivo nucleosome occupancies at functional and nonfunctional sites at selected loci of each class. For ease of comparison, we highlighted the predicted TFBSs and the corresponding occupancies in grey. It should also be noted that the peaks from ChIP-seq data or nucleosome occupancy data do not need to align with each other to indicate overlap of TFs and nucleosomes for two reasons: (1) a TF binding anywhere along the 147bp of nucleosome DNA will influence nucleosome and TF interactions and (2) the peaks of ChIP-seq data do not necessarily align with the binding motif, due to resolution that is limited by the size of sonicated fragments.

For class 1 genes, in vitro and in vivo nucleosome occupancies were often correlated with each other at both functional and non-functional sites, as shown in Fig 3b and 3c. In addition, we observed that nucleosome occupancies for non-functional sites seem to be lower than at functional sites. We next examined class 2 genes. Unlike class 1 genes, class 2 functional Oct4/Sox2 binding sites exhibit different nucleosome occupancies in vitro and in vivo (Fig 3d and 3e), Comparing to the in vitro occupancy, nucleosomes appeared to be depleted in vivo at functional sites but not at non-functional sites in class 2 genes. Lastly, we examined class 3 genes as a different set of poised genes lacking functional Oct4/Sox2 sites. No depletion of in vivo occupancy compared to in vitro at non-functional sites was seen. Taken together, by examining in vitro and in vivo nucleosome occupancy tracks of at TBFS in distinct gene sets, it appears that the functional sites for Oct4/Sox2 transcription factors can have different relationships to nucleosome occupancy.

### Differential nucleosome occupancies at functional Sox2/Oct4 binding sites

To confirm the trends observed in the UCSC genome browser tracks, we conducted a quantitative analysis of the normalized, GC-adjusted nucleosome occupancy scores for in vivo and in vitro datasets. The in vivo and in vitro occupancy at each predicted site was determined by averaging the adjusted occupancy across the 15bp binding site. The fold-change between in vivo and in vitro nucleosome occupancy at each site was calculated by taking the log_2_ of the ratio of in vivo occupancy over in vitro occupancy. We then examined how nucleosome occupancy and fold-change varied across functional TFBS at the different defined gene classes.

**Fig 4:**
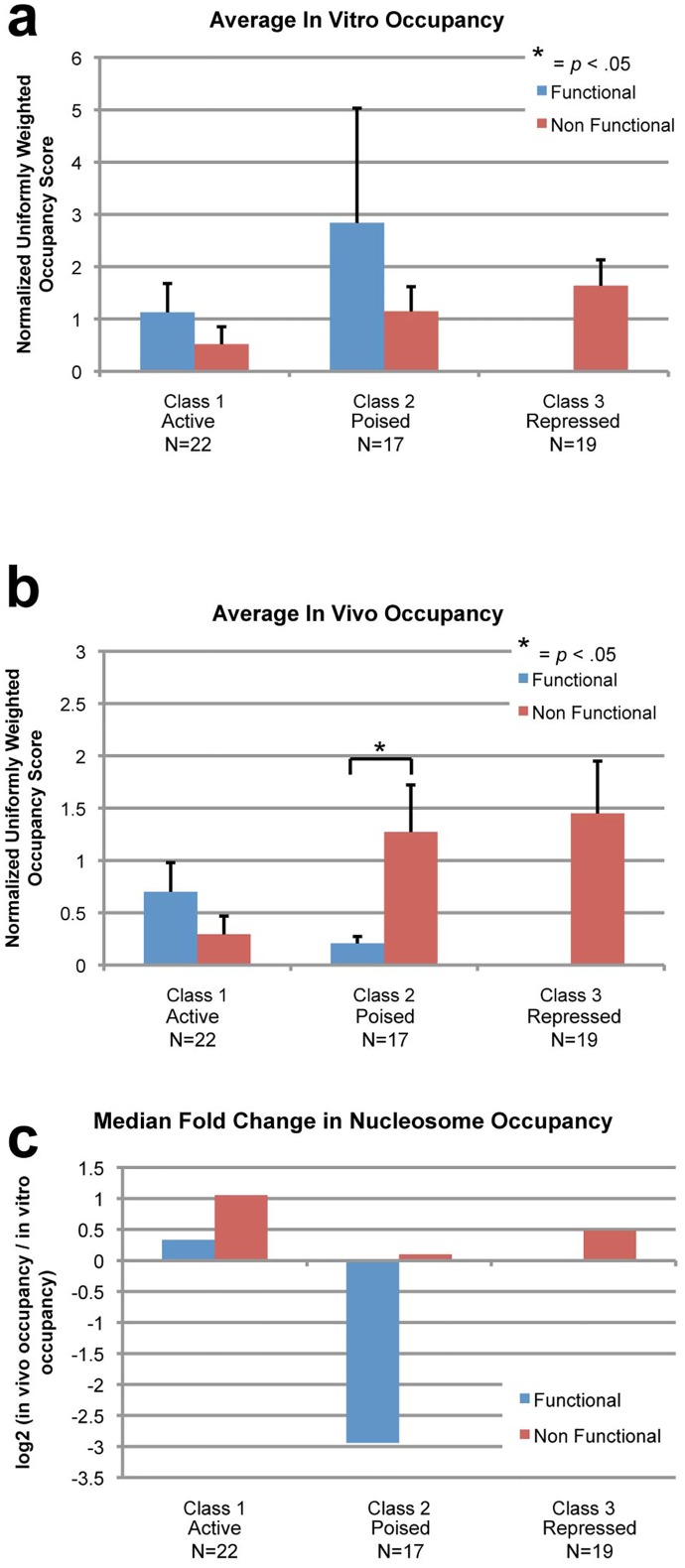
Correlations between functional transcription factor binding and nucleosome occupancies. Using the average transcription factor (TF) occupancy (Oct4 and Sox2, from Whyte *et al*.) across the 15bp predicted binding sites for Oct4 and Sox2 (n = 58), we identified each site as functional or nonfunctional using a TF occupancy score of > = 20 as a cutoff. We also separated each set of factors by the type of downstream gene regulation (Class 1 Active: functional (n = 10) and nonfunctional (n = 12), Class 2 Poised: functional (n = 4) and nonfunctional (n = 13), Class 3 Repressed: nonfunctional (n = 19)). We calculated the in vitro and in vivo occupancy over the 15bp of each binding site and found the average of each gene type and binding site type. We also calculated the log_2_ of the ratio of in vivo to in vitro occupancy at each binding site and found the median for each gene and site type. The standard error of the mean is displayed in the error bars. Paired Z-scores between different classes were calculated and p-values < 0.05 are marked with an asterisk. (a) In vitro occupancy is not significantly different at functional versus nonfunctional sites for both class 1 and class 2 genes. (b) At class 1 genes, in vivo occupancy is not significantly different at functional TFBS. At class 2 genes however, in vivo occupancy is significantly lower at functional TFBS. (c) For class 1 genes’ functional sites, the median fold-change is small and positive, while at class 2 the median fold-change is large and negative. Nonfunctional sites across all gene classes were positive.

For class 1 genes, we found no significant differences of in vivo or in vitro nucleosome occupancy between functional and nonfunctional sites (Fig 4a and 4b). We also found that the median fold-change for both functional and nonfunctional sites was small (Fig 4c). However, class 2 genes saw different distributions of nucleosome occupancies. While there was no significant difference of in vitro occupancy between functional and nonfunctional sites (Fig 4a), in vivo occupancy was significantly lower at functional sites (Fig 4b). Additionally, the median fold-change was also much larger and negative at functional sites (Fig 4c). Class 3 genes only contain nonfunctional sites, and did not have significantly different nucleosome occupancy or fold change compared to other nonfunctional sites (Fig 4a–4c).

## Discussion

The advent of ChIP-seq technology greatly facilitated the identification of functional transcription factor binding sites within the genome. However, it does not reveal the underlying mechanism of why these sites, over the many other possible sites within the genome, are functional. As a step to understand the molecular basis for functional transcription factor binding sites, we examined the interplay between predicted transcription factor sites, functional transcription factor sites, and the underlying nucleosome occupancy. Our study, albeit at a limited scale, allowed us to examine the nucleosome landscape for several promoters with great detail. Using the first experimentally derived map of in vitro nucleosome occupancy for mouse ESCs, in addition to in vivo occupancy maps, we identified two distinct patterns of nucleosome occupancy over functional binding sites in the Oct4/Sox2 network, suggesting that Oct4 and Sox2 binding can differentially affect nucleosome occupancy at TFBS.

Since histone octamer proteins cover such a large percentage of the genome, nucleosomes and transcription factors compete for access to potential TF binding sites. Without competition from TFs, we might predict that areas with high intrinsic nucleosome occupancy would be unlikely to contain functional binding sites, while areas of low intrinsic nucleosome occupancy would be more likely to contain functional sites, since this arrangement would seem most energetically favorable [50]. However, in our examination of the Oct4/Sox2 transcriptional network, we found that functional TFBS are not depleted of in vitro occupancy. This result agrees with studies that have found correlations between TF occupancy and in vitro occupancy in higher eukaryotes [31, 33, 51]. Furthermore, the nonfunctional TF sites are not in higher areas of in vitro occupancy, yet are not utilized. Therefore, our results do not provide evidence that high nucleosome occupancy is used to mask nonfunctional TFBS, suggesting that other mechanisms may be employed.

When we examined nucleosome occupancy for our class 1 genes, Oct4 and Sox, in vivo nucleosome occupancy was not significantly different at functional TFBS than at nonfunctional, suggesting little displacement of in vivo nucleosomes versus in vitro. Recent studies have revealed instances of TFs binding to stretches of DNA that are already occupied by a nucleosome, and refer to these factors as pioneer factors [52–54]. Overlap of TFs and in vivo nucleosome occupancy at Oct4 and Sox2 genes in our experiments would be consistent with Oct4 and Sox2 acting as pioneer transcription factors at certain loci, including their own promoters [43, 55, 56]. However, without an experiment such as a sequential histone/TF CHiP we must also consider the possibility that there are two populations of cells, one with TF bound and one with nucleosomes bound. At the Pax6 and Nestin loci, which are class 2 genes that are poised with bivalent post-translational histone modifications in embryonic stem cells [41], functional sites were significantly correlated with depleted levels of in vivo occupancy, compared to both in vitro occupancy as well as occupancy at nonfunctional sites. This is in sharp contrast to the fold-change observed at functional sites at class 1 genes. This suggests the possibility that Oct4 and Sox2 could interact with chromatin differently at different locations.

It has been established that pluripotent transcription factors such as Oct4/Sox2 bind to both transcriptionally active and poised genes in ES cells and regulate downstream targets in different ways [57, 58]. Intriguingly, we find that functional transcription factor sites are located within different nucleosome organizations, which correspond to these different categories of genes in our experiments. Hence, we hypothesize that transcription factors, such as Oct4/Sox2, may be able to distinguish between functions by binding to TFBS with different nucleosome occupancies. When Oct4/Sox2 is regulating genes in an activating manner, TFs and nucleosomes may co-occupy at functional TFBSs. However, when Oct4/Sox2 is regulating poised genes, Oct4/Sox2 might displace nucleosomes at functional sites. Future experiments to generate high-coverage genome-wide datasets of in vitro nucleosome maps in combination with in vivo maps will be necessary to further examine these hypotheses.

## Supporting Information

S1 File Supporting information for embryonic stem cell culture, JASPAR prediction, and data summary. The quality of embryonic stem cell cultures was monitored by quantification of differentiation using florescent staining and ImageJ analysis (Figure A). The nucleosome repeat length of embryonic stem cells was also measured via micrococcal nuclease digestion (Figure B). The JASPAR matrix and regions scanned to find predicted Oct4/Sox2 binding sites are presented in Figure C and the results of the analysis are presented in Table A. The quantification of TF occupancy, in vivo nucleosome occupancy, and in vitro nucleosome occupancy for each predicted binding site are summarized in Table B.
(DOCX)
