# Supplementary material for: Differential Nucleosome Occupancies across Oct4-Sox2 Binding Sites in Murine Embryonic Stem Cells
Source: PLoS One. 2015 May 18;10(5):e0127214. doi: 10.1371/journal.pone.0127214 (PMC4436218; doi:10.1371/journal.pone.0127214)
Supplement: S1 File — The quality of embryonic stem cell cultures was monitored by quantification of differentiation using florescent staining and ImageJ analysis (Figure A). The nucleosome repeat length of embryonic stem cells was also measured via micrococcal nuclease digestion (Figure B). The JASPAR matrix and regions scanned to find predicted Oct4/Sox2 binding sites are presented in Figure C and the results of the analysis are presented in Table A. The quantification of TF occupancy, in vivo nucleosome occupancy, and in vitro nucleosome occupancy for each predicted binding site are summarized in Table B. (DOCX) [file pone.0127214.s001.docx]

**Supporting Information**

**
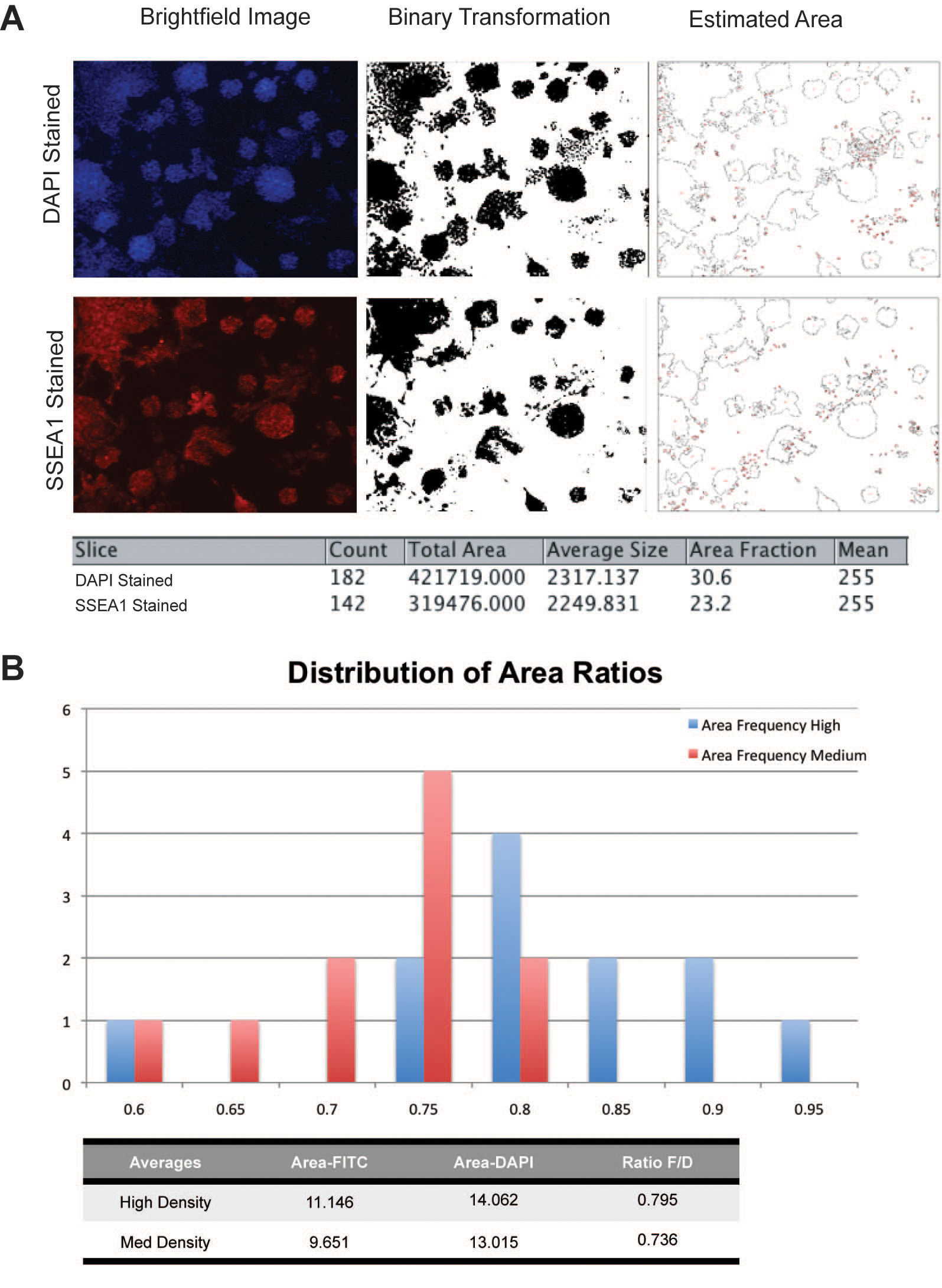
**

**Figure A. Quantification of Differentiation in Embryonic Stem Cell Cultures.**

(a) Cells were seeded at medium (2.0 x 10^4^ cells/cm^2^) and high (2.5 x 10^4^ cells/cm^2^) densities, then examined to determine the average amount of non-differentiated cells in each condition. Cells were stained with 1:500 DAPI in PBS as well as probed with 1:50 dilution SSEA1 antibody in 5% NGS (Millipore MAB4301) followed by 1:200 dilution of Rhodamine secondary antibody in 5% NGS. They were then fluorescently imaged and analyzed with ImageJ. The area of the cells was calculated by converting the images to black and white using the Threshold tool, then using the Analyze Particle feature with the minimum pixel size set to 20. (b) The ratio of SSEA1-stained area to DAPI-stained area was used as a measure of non-differentiated cells. The graph shows that the highest density seeding results in the most non-differentiated cells, on average 80%.


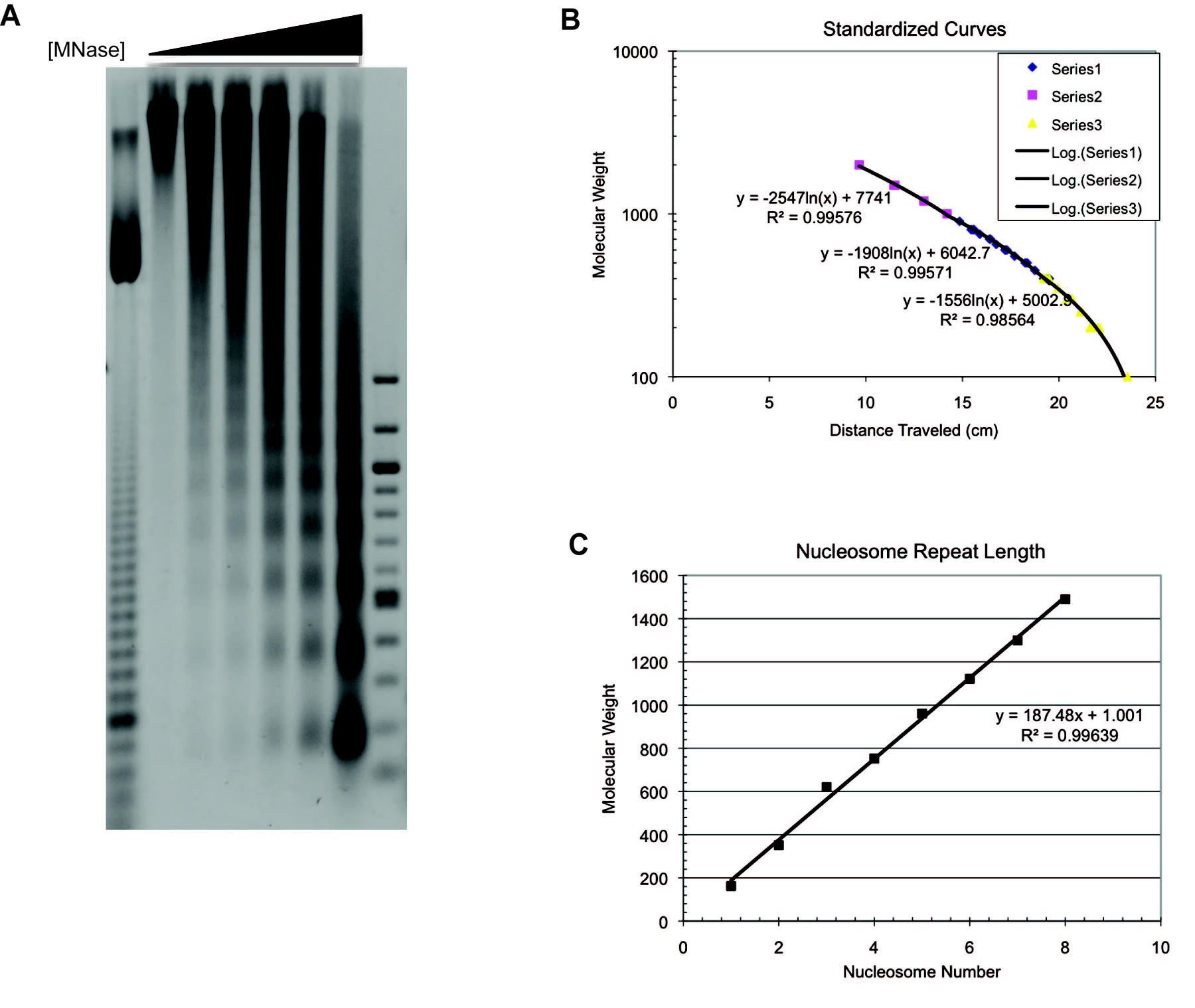


**Figure B. Nucleosome Repeat Length of Embryonic Stem Cells.**

(a) Low levels of MNase digestion (Lanes 2-7: 25 U*min/mL to 500 U*min/mL, Lane 1: 50bp ladder, Lane 8: 1kb ladder) were used to calculate the nucleosome repeat length of embryonic stem cells. (b) The distance migrated for each molecular weight standard was plotted against its known molecular weight on a log-scale. Lines of best fit were then calculated for three regimes; low, medium, and high molecular weight. The distance of each nucleosomal band in the last digested lane was measured and the molecular weight was found using the appropriate best fit formula. (c) The number of nucleosomes contained in each band was plotted against their molecular weight linearly. The slope of this line is the nucleosome repeat length, which is 187 bp. This corresponds to a linker length of 40 bp.


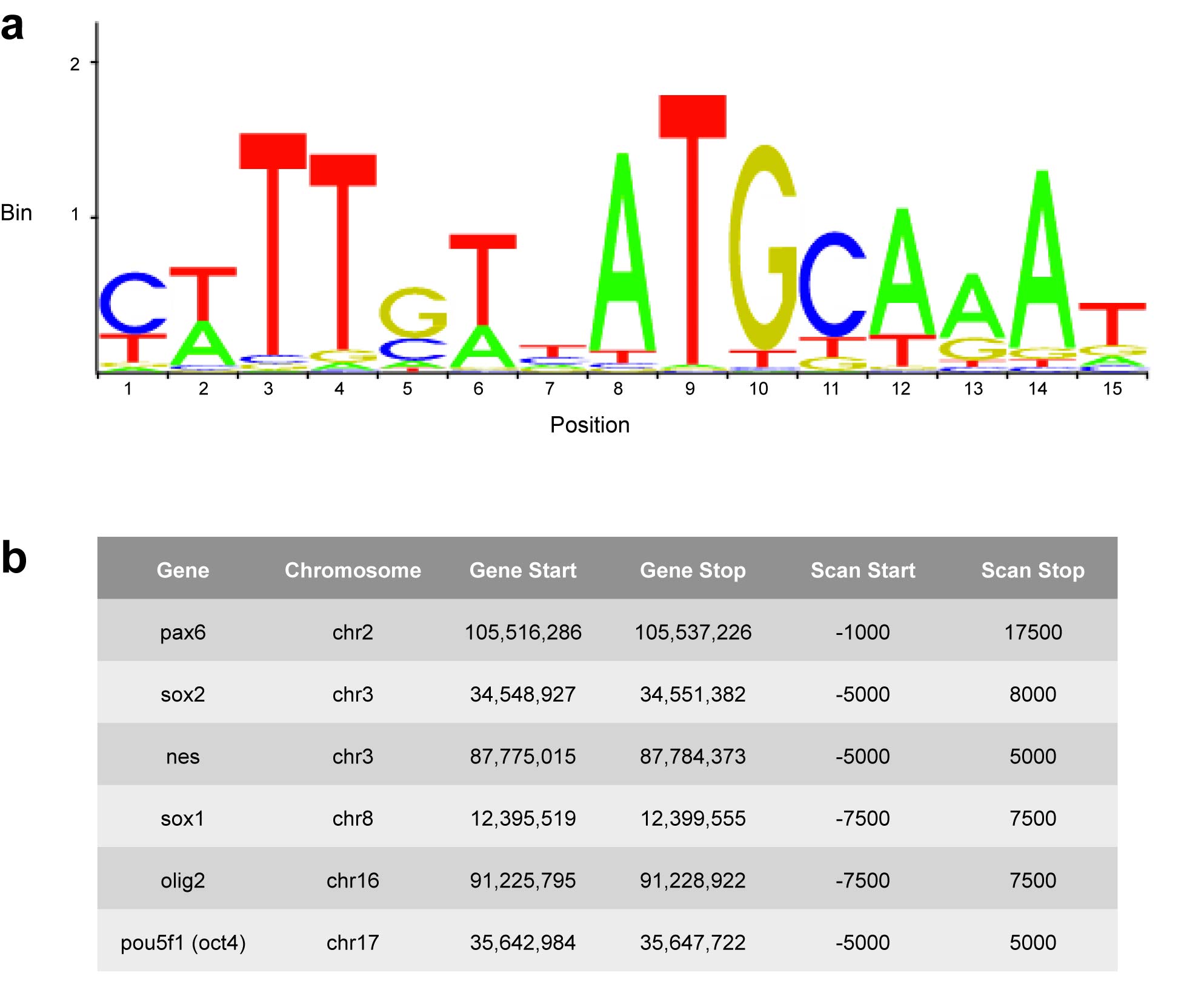


**Figure C. Prediction of Oct4/Sox2 Binding Sites Using JASPAR.**

(a) The binding matrix for Oct4 and Sox2 concurrent binding, from the JASPAR database. (b) This table highlights the regions that were scanned for predicted binding sites. The scan start and scan stop are the number of bps upstream (negative) or downstream (positive) from the gene start site.

**Table A. JASPAR Transcription Factor Binding Site Predictions for Oct4:Sox2.**

| Chromosome | Site Start | Site End | Site Strength | Strand |
| --- | --- | --- | --- | --- |
| chr2 | 105515410 | 105515424 | 9.3 | - |
| chr2 | 105520726 | 105520740 | 13.6 | + |
| chr2 | 105522703 | 105522717 | 12.7 | + |
| chr2 | 105525049 | 105525063 | 13 | + |
| chr2 | 105527040 | 105527054 | 9.4 | - |
| chr2 | 105529218 | 105529232 | 10 | - |
| chr2 | 105530916 | 105530930 | 9.8 | + |
| chr2 | 105531586 | 105531600 | 12.3 | - |
| chr2 | 105531791 | 105531805 | 11.9 | + |
| chr2 | 105532141 | 105532155 | 12.5 | + |
| chr2 | 105532750 | 105532764 | 10.6 | + |
| chr2 | 105533203 | 105533217 | 8.8 | + |
| chr2 | 105533618 | 105533632 | 10.3 | + |
| chr3 | 34545124 | 34545138 | 9.8 | + |
| chr3 | 34545131 | 34545145 | 11 | - |
| chr3 | 34545340 | 34545354 | 12.6 | + |
| chr3 | 34545593 | 34545607 | 10.3 | - |
| chr3 | 34545624 | 34545638 | 9.4 | + |
| chr3 | 34546024 | 34546038 | 10.8 | + |
| chr3 | 34546151 | 34546165 | 9.5 | + |
| chr3 | 34546622 | 34546636 | 9.6 | - |
| chr3 | 34551512 | 34551526 | 9.6 | - |
| chr3 | 34552982 | 34552996 | 17.5 | + |
| chr3 | 34553293 | 34553307 | 9.7 | - |
| chr3 | 34553508 | 34553522 | 10.4 | - |
| chr3 | 34553527 | 34553541 | 9.2 | + |
| chr3 | 34555220 | 34555234 | 10.6 | - |
| chr3 | 87771808 | 87771822 | 9.7 | + |
| chr3 | 87772832 | 87772846 | 10.1 | + |
| chr3 | 87772838 | 87772852 | 10.4 | + |
| chr3 | 87778433 | 87778447 | 15 | - |
| chr8 | 12390436 | 12390450 | 9.5 | + |
| chr8 | 12391923 | 12391937 | 9.5 | + |
| chr8 | 12392389 | 12392403 | 9.1 | + |
| chr8 | 12392441 | 12392455 | 9.5 | + |
| chr8 | 12392632 | 12392646 | 16.2 | + |
| chr8 | 12392738 | 12392752 | 11.1 | + |
| chr8 | 12398307 | 12398321 | 10.1 | - |
| chr8 | 12399416 | 12399430 | 11.5 | + |
| chr8 | 12400796 | 12400810 | 8.9 | + |
| chr8 | 12401951 | 12401965 | 11.2 | - |
| chr8 | 12402766 | 12402780 | 9.1 | + |
| chr16 | 91218454 | 91218468 | 9.7 | + |
| chr16 | 91218467 | 91218481 | 11.5 | + |
| chr16 | 91218502 | 91218516 | 13.7 | - |
| chr16 | 91218753 | 91218767 | 10.3 | + |
| chr16 | 91219673 | 91219687 | 9.2 | + |
| chr16 | 91225200 | 91225214 | 12.7 | + |
| chr16 | 91228704 | 91228718 | 11.5 | - |
| chr16 | 91231953 | 91231967 | 12.3 | - |
| chr17 | 35639543 | 35639557 | 8.7 | + |
| chr17 | 35640979 | 35640993 | 14.6 | - |
| chr17 | 35641180 | 35641194 | 9.2 | - |
| chr17 | 35641699 | 35641713 | 13.5 | + |
| chr17 | 35643599 | 35643613 | 10.8 | + |
| chr17 | 35644349 | 35644363 | 10.5 | + |
| chr17 | 35644889 | 35644903 | 8.7 | - |
| chr17 | 35646906 | 35646920 | 9.2 | + |

**Table B. Averaged Occupancies of Oct4, Sox2, In Vivo Nucleosome and In Vitro Nucleosomes Across Predicted Transcription Factor Binding Sites.**

| Chromosome | Gene | Class | Site Start | Site End | Avg Oct4 Occ | Avg Sox2 Occ | Avg In vivo Occ | Avg In vitro occ | Functional |
| --- | --- | --- | --- | --- | --- | --- | --- | --- | --- |
| Class 1 | sox2 | chr3 | 34545593 | 34545607 | 18 | 4 | 0.11 | 0.01 | n |
| Class 1 | sox2 | chr3 | 34545624 | 34545638 | 18 | 4 | 0.08 | 0.01 | n |
| Class 1 | sox2 | chr3 | 34546024 | 34546038 | 5 | 5 | 0.08 | 0.01 | n |
| Class 1 | sox2 | chr3 | 34546151 | 34546165 | 8 | 4 | 0.74 | 2.80 | n |
| Class 1 | sox2 | chr3 | 34546622 | 34546636 | 9 | 2 | 0.14 | 0.01 | n |
| Class 1 | sox2 | chr3 | 34551512 | 34551526 | 5 | 0 | 0.01 | 0.01 | n |
| Class 1 | sox2 | chr3 | 34553293 | 34553307 | 13 | 13 | 0.16 | 0.01 | n |
| Class 1 | sox2 | chr3 | 34553508 | 34553522 | 9 | 1 | 0.01 | 0.01 | n |
| Class 1 | sox2 | chr3 | 34553527 | 34553541 | 9 | 1 | 0.02 | 0.01 | n |
| Class 1 | oct4_ | chr17 | 35643599 | 35643613 | 14 | 1 | 2.29 | 3.60 | n |
| Class 1 | oct4_ | chr17 | 35644349 | 35644363 | 8 | 3 | 0.18 | 0.01 | n |
| Class 1 | oct4_ | chr17 | 35644889 | 35644903 | 10 | 2 | 0.01 | 0.14 | n |
| Class 1 | sox2 | chr3 | 34545124 | 34545138 | 28 | 19 | 0.21 | 0.01 | y |
| Class 1 | sox2 | chr3 | 34545131 | 34545145 | 28 | 19 | 0.17 | 0.01 | y |
| Class 1 | sox2 | chr3 | 34545340 | 34545354 | 308 | 155 | 0.10 | 0.01 | y |
| Class 1 | sox2 | chr3 | 34552982 | 34552996 | 388 | 240 | 0.01 | 0.01 | y |
| Class 1 | sox2 | chr3 | 34555220 | 34555234 | 59 | 14 | 0.18 | 0.14 | y |
| Class 1 | oct4_ | chr17 | 35639543 | 35639557 | 20 | 23 | 1.13 | 5.64 | y |
| Class 1 | oct4_ | chr17 | 35640979 | 35640993 | 250 | 137 | 0.46 | 1.94 | y |
| Class 1 | oct4_ | chr17 | 35641180 | 35641194 | 72 | 63 | 0.29 | 1.21 | y |
| Class 1 | oct4_ | chr17 | 35641699 | 35641713 | 34 | 18 | 1.81 | 1.46 | y |
| Class 1 | oct4_ | chr17 | 35646906 | 35646920 | 23 | 4 | 2.63 | 0.87 | y |
| Class 2 | nes | chr3 | 87771808 | 87771822 | 2 | 3 | 5.41 | 5.13 | n |
| Class 2 | nes | chr3 | 87772832 | 87772846 | 2 | 6 | 2.20 | 1.56 | n |
| Class 2 | nes | chr3 | 87772838 | 87772852 | 2 | 6 | 1.56 | 1.45 | n |
| Class 2 | pax6 | chr2 | 105520726 | 105520740 | 13 | 5 | 0.01 | 0.01 | n |
| Class 2 | pax6 | chr2 | 105522703 | 105522717 | 2 | 1 | 0.35 | 0.89 | n |
| Class 2 | pax6 | chr2 | 105525049 | 105525063 | 4 | 7 | 0.30 | 0.15 | n |
| Class 2 | pax6 | chr2 | 105527040 | 105527054 | 5 | 2 | 0.28 | 0.98 | n |
| Class 2 | pax6 | chr2 | 105529218 | 105529232 | 3 | 2 | 0.01 | 0.01 | n |
| Class 2 | pax6 | chr2 | 105530916 | 105530930 | 4 | 2 | 0.12 | 0.01 | n |
| Class 2 | pax6 | chr2 | 105532141 | 105532155 | 13 | 8 | 0.24 | 0.01 | n |
| Class 2 | pax6 | chr2 | 105532750 | 105532764 | 14 | 7 | 3.04 | 4.33 | n |
| Class 2 | pax6 | chr2 | 105533203 | 105533217 | 4 | 3 | 0.59 | 0.01 | n |
| Class 2 | pax6 | chr2 | 105533618 | 105533632 | 2 | 1 | 2.45 | 0.40 | n |
| Class 2 | nes | chr3 | 87778433 | 87778447 | 269 | 125 | 0.35 | 9.42 | y |
| Class 2 | pax6 | chr2 | 105515410 | 105515424 | 25 | 4 | 0.04 | 0.47 | y |
| Class 2 | pax6 | chr2 | 105531586 | 105531600 | 172 | 106 | 0.26 | 0.61 | y |
| Class 2 | pax6 | chr2 | 105531791 | 105531805 | 31 | 17 | 0.18 | 0.85 | y |
| Class 3 | sox1 | chr8 | 12390436 | 12390450 | 7 | 2 | 0.20 | 0.84 | n |
| Class 3 | sox1 | chr8 | 12391923 | 12391937 | 15 | 0 | 3.05 | 6.82 | n |
| Class 3 | sox1 | chr8 | 12392389 | 12392403 | 3 | 4 | 0.51 | 0.01 | n |
| Class 3 | sox1 | chr8 | 12392441 | 12392455 | 3 | 4 | 0.32 | 0.01 | n |
| Class 3 | sox1 | chr8 | 12392632 | 12392646 | 2 | 1 | 0.15 | 0.01 | n |
| Class 3 | sox1 | chr8 | 12392738 | 12392752 | 3 | 4 | 0.21 | 0.01 | n |
| Class 3 | sox1 | chr8 | 12398307 | 12398321 | 5 | 5 | 0.01 | 0.01 | n |
| Class 3 | sox1 | chr8 | 12399416 | 12399430 | 5 | 0 | 0.02 | 0.01 | n |
| Class 3 | sox1 | chr8 | 12400796 | 12400810 | 13 | 6 | 0.32 | 0.79 | n |
| Class 3 | sox1 | chr8 | 12401951 | 12401965 | 10 | 1 | 4.39 | 5.73 | n |
| Class 3 | sox1 | chr8 | 12402766 | 12402780 | 9 | 9 | 1.01 | 0.14 | n |
| Class 3 | olig2 | chr16 | 91218454 | 91218468 | 10 | 6 | 0.60 | 2.97 | n |
| Class 3 | olig2 | chr16 | 91218467 | 91218481 | 10 | 6 | 0.33 | 1.69 | n |
| Class 3 | olig2 | chr16 | 91218502 | 91218516 | 10 | 2 | 0.39 | 0.05 | n |
| Class 3 | olig2 | chr16 | 91218753 | 91218767 | 2 | 3 | 0.76 | 1.90 | n |
| Class 3 | olig2 | chr16 | 91219673 | 91219687 | 3 | 0 | 6.69 | 3.31 | n |
| Class 3 | olig2 | chr16 | 91225200 | 91225214 | 25 | 6 | 1.05 | 1.64 | n |
| Class 3 | olig2 | chr16 | 91228704 | 91228718 | 5 | 1 | 6.91 | 4.95 | n |
| Class 3 | olig2 | chr16 | 91231953 | 91231967 | 1 | 4 | 0.65 | 0.23 | n |
